# Trends in respiratory disease mortality in the United States, 1999–2024

**DOI:** 10.3389/fmed.2026.1879935

**Published:** 2026-07-23

**Authors:** Wenjing Wu, Ruonan Tian, Huaqing Guo, Zhiguang Duan

**Affiliations:** School of Humanities and Social Sciences, Shanxi Medical University, Taiyuan, China

**Keywords:** mortality, region, respiratory disease, sex, trends

## Abstract

**Background:**

Respiratory diseases are a leading cause of death in the US. This study examines mortality trends from 1999 to 2024 using CDC WONDER data, focusing on disparities by sex, age, race/ethnicity, and region.

**Methods:**

An ecological time-trend study calculated crude and age-adjusted mortality rates (AAMR) and used Joinpoint regression to estimate annual changes.

**Results:**

From 1999 to 2024, respiratory disease deaths increased from 227,985 to 263,209 (+15.5%), while AAMR declined from 129.37 to 91.70 per 100,000 (AAPC = −1.37). The decline accelerated after 2018. AAMR was consistently higher in males, but decreased more steeply in males. All regions showed declining AAMR, with the West having the largest reduction, whereas the South had the largest rise in death counts. All racial/ethnic groups saw AAMR declines, yet non-Hispanic Whites bore the highest burden, and Hispanics/other races showed larger absolute death increases. Older adults carried the heaviest burden, while adults aged 25–34 years experienced a slight AAMR increase.

**Conclusion:**

Although age-adjusted respiratory mortality declined continuously from 1999 to 2024, the absolute number of deaths rose, with marked subgroup heterogeneity. Future strategies should target men, older adults, Southern residents, and younger adults.

## 1 Introduction

Respiratory diseases, as a major global threat to health, encompass a wide spectrum of pathological entities, including chronic obstructive pulmonary disease (COPD), asthma, pneumonia, and acute lower respiratory tract infections. These conditions are characterized by high prevalence, substantial disability, and an elevated risk of mortality, imposing a considerable burden on both individual quality of life and healthcare systems. According to data from the World Health Organization, respiratory diseases are the third leading cause of death worldwide, ranking only behind cardiovascular diseases and cancers (1). The Global Burden of Disease Study 2019 (GBD 2019) reported that COPD and lower respiratory tract infections alone accounted for approximately 3.5 million deaths globally (2). In China, although the standardized mortality rate of chronic respiratory diseases has shown a downward trend, it remains a major public health concern (3). In the United States, respiratory diseases likewise occupy a prominent position among leading causes of death. Data from the Centers for Disease Control and Prevention (CDC) indicate that respiratory diseases are the fifth leading cause of death in the country (4). From 2022 to 2023, the age-adjusted death rate for chronic lower respiratory diseases decreased by 2.6% (from 34.3 to 33.4) (5). Among these, COPD and pneumonia rank as the sixth and ninth leading causes of death in the United States, respectively. This high lethality stems not only from the intrinsic pathological complexity of these conditions, but is also closely linked to population aging, ambient air pollution, and pronounced socioeconomic inequalities (6). Therefore, this study aims to characterize mortality from respiratory diseases in the United States from 1999 to 2024 and to elucidate disparities across age, sex, geographic regions, and racial/ethnic groups. By identifying key inflection points in mortality trends, we further explore potential drivers underlying these temporal changes.

## 2 Methods

### 2.1 Data sources

This study utilized data from the CDC Wide-ranging Online Data for Epidemiologic Research (CDC WONDER) mortality database for the period 1999–2024. All deaths attributable to respiratory diseases among adults aged 25 years and older were included in the analysis. Respiratory disease–related deaths were identified using the International Classification of Diseases, Tenth Revision (ICD − 10) codes J00–J98 as the underlying cause of death. Data on age, race/ethnicity, sex, year, Census division, region, and state were extracted. Race/ethnicity was categorized as Hispanic, non-Hispanic White, non-Hispanic Black, and non-Hispanic individuals of other races, and Census regions were grouped into Northeast, Midwest, South, and West.

Data for 1999–2020 were obtained from the “Underlying Cause of Death, 1999–2020” dataset, and data for 2021–2024 were extracted from the “Underlying Cause of Death, 2018–2024” dataset. To ensure temporal comparability, all age-adjusted mortality rates were calculated using the 2000 U.S. standard population.

To accommodate the transition in racial and ethnic classification standards from bridged-race to single-race population estimates implemented by the NCHS in 2021 (7), mortality data for 1999–2020 were extracted for 1999–2020 from the Underlying Cause of Death, 1999–2020 dataset using bridged-race categories, and data for 2018–2024 using single-race categories, with 2018–2020 overlapping between the two datasets (8). Previous studies at the national level have suggested that the use of single-race, as opposed to bridged-race, categories has minimal impact on age-adjusted mortality rates for all major racial and ethnic groups, and the CDC WONDER results for 2018–2020 are consistent across the two datasets (9). Therefore, in this study we used data for 2019–2020 from the Underlying Cause of Death, 1999–2020 dataset.

Deaths due to COVID−19 are classified under code U07.1 and are therefore not included in the J00–J98 category. Although COVID−19 affects the respiratory system, its systemic nature, high transmissibility, and specific control measures distinguish it from typical J00–J98 respiratory diseases, and separate analysis is more appropriate for public health surveillance purposes. Moreover, the present study aims to examine the impact of traditional respiratory diseases on mortality; incorporating U07.1 could compromise the temporal homogeneity of the time series. For these reasons, deaths attributable to COVID-19 were not included in the analysis.

### 2.2 Research methods

To examine national trends in mortality from respiratory diseases, we used the Joinpoint Regression Program to calculate the crude mortality rates and age-adjusted mortality rates (AAMR) per 100,000 population from 1999 to 2024, stratified by age, race/ethnicity, sex, and Census region, and reported the corresponding 95% confidence intervals. Data analysis was conducted from December 2025 to March 2026. Statistical analyses were performed using R Studio (version 2025.09.2, Build 418; Posit Software, PBC, 2025) and the Joinpoint Regression Program (version 5.4.0.0, National Cancer Institute, 2025). The Joinpoint model was used to fit temporal trends and detect statistically significant changes in these trends.

A grid search method was applied to identify significant changes in temporal trends. The number of joinpoints was allowed to vary from 0 to 4, with a minimum of two observations required before the first joinpoint, after the last joinpoint, and between adjacent joinpoints. The optimal model was selected using the Monte Carlo permutation test with 4,499 permutations and an overall significance level of 0.05. Annual percent change (APC) and average annual percent change (AAPC) with corresponding 95% confidence intervals (CIs) were calculated to quantify temporal changes in mortality rates.

Based on the univariate analysis, we extended the decomposition analysis by stratifying the population by both sex and U.S. Census region (Northeast, Midwest, South, and West). For each sex-region subgroup, total mortality was expressed as the sum of age-specific population proportions and mortality rates (10). Changes in mortality between 1999 and 2024 were decomposed into age effects and rate effects using a standard demographic decomposition approach (Equation 1). This approach allowed us to quantify how the contributions of population aging and mortality rate changes varied across both sex and geographic regions (11). This part of the analysis was conducted using R Studio software.


$$\Delta M_{g,r}=\sum (P_{a,2024}-P_{a,1999})r_{a,\mathrm{avg}}+\sum (r_{a,2024}-r_{a,1999})P_{a,\mathrm{avg}}$$ (1)

Let *a* denote age group, *g* denote sex, and *r* denote Census region. *P*(*a*,*t*) represents the proportion of the population in age group *a* at year *t*, and *r*(*a*,*t*) represents the age-specific mortality rate for age group *a* at year *t*. The first term (age effect) quantifies the contribution of population aging, whereas the second term (rate effect) quantifies the contribution of changes in age-specific mortality rates.

## 3 Results

From 1999 to 2024, a total of 7,273,625 deaths from respiratory diseases were recorded, representing a 15.45% increase in the number of deaths attributable to respiratory conditions (Table 1). Of these, 3,526,595 (48.48%) occurred in males. Overall, while the absolute number of deaths due to respiratory diseases showed an upward trend, the age-standardized mortality rate declined substantially over the study period. The AAMR in 1999 was 129.37 deaths per 100,000 population (95% CI: 128.84–129.90), declining to 91.70 deaths per 100,000 population by 2024 (95% CI: 91.34–92.05). Over the study period, the total number of deaths among males (3,526,595) was lower than among females (3,747,030), yet the relative increase in mortality was greater in males. By U.S. Census region, the South recorded the highest number of deaths (2,823,132) and the largest increase in mortality rates. With respect to race/ethnicity, non-Hispanic White individuals had both the highest absolute number of deaths (6,172,849) and the greatest increase in mortality rates. The largest number of deaths occurred in the 75–84-year age group (2,115,967), whereas the greatest increase in mortality rates was observed in those aged 55–64 years. Notably, the age-adjusted mortality rate was highest among individuals aged 25–34 years.

**Table 1:** Respiratory diseases deaths-and AAMR in-1999-and-2024.

Characteristic	Deaths			AAMR		
	1999	2024	Percent change (%)	1999 (95% CI)	2024 (95% CI)	AAPC (95% CI)
Overall	227,985	263,209	15.45	129.37 (128.84 to 129.90)	91.70 (91.34 to 92.05)	−1.37 (−1.65 to −1.09)*
Sex						
Male	110,218	129,668	17.65	165.44 (164.44 to 166.43)	103.36 (102.79 to 103.93)	−1.91 (−2.19 to −1.63)*
Female	117,767	133,541	13.39	108.25 (107.63 to 108.87)	82.87 (82.42 to 83.32)	−1.13 (−1.44 to −0.82)*
Census region						
Northeast	45,931	44,724	−2.63	122.13 (121.01 to 123.25)	84.39 (83.61 to 85.19)	−1.57 (−2.14 to −0.99)*
Midwest	56,384	60,232	6.82	132.80 (131.71 to 133.90)	99.86 (99.06 to 100.67)	−1.04 (−1.39 to −0.69)*
South	82,781	107,400	29.74	134.39 (133.47 to 135.31)	98.24 (97.65 to 98.84)	−1.20 (−1.46 to −0.95)*
West	42,889	50,853	18.57	123.87 (122.70 to 125.05)	78.84 (78.15 to 79.53)	−1.77 (−2.65 to −0.89)*
Race						
Hispanic	6,450	15,786	144.74	83.21 (81.09 to 85.32)	54.79 (53.92 to 55.67)	−1.69 (−1.84 to −1.55)*
NH Black	16,990	23,676	39.35	115.76 (114.00 to 117.53)	84.40 (83.30 to 85.51)	−1.32 (−1.75 to −0.89)*
NH White	200,551	213,919	6.67	134.03 (133.44 to 134.61)	101.92 (101.48 to 102.36)	−1.11 (−1.40 to −0.81)*
NH Other	3,268	8,945	173.71	77.34 (74.57 to 80.11)	43.89 (42.98 to 44.82)	−2.43 (−2.75 to −2.11)*
Age^#^						
25–34 years	1,008	1,255	24.50	2.51 (2.51 to 2.51)	2.70 (2.70 to 2.70)	0.66 (0.03 to 1.29)*
35–44 years	2,713	2,594	−4.39	6.02 (6.02 to 6.02)	5.70 (5.70 to 5.70)	−0.03 (−0.43 to 0.37)
45–54 years	6,340	6,495	2.44	17.33 (17.33 to 17.33)	15.93 (15.93 to 15.93)	−0.38 (−1.05 to 0.30)
55–64 years	16,846	26,430	56.89	70.85 (70.85 to 70.85)	63.44 (63.44 to 63.44)	−0.47 (−0.93 to 0.00)*
65–74 years	46,977	63,658	35.51	255.05 (255.05 to 255.05)	179.60 (179.60 to 179.60)	−1.44 (−1.75 to −1.13)*
75–84 years	82,113	87,823	6.95	671.69 (671.69 to 671.69)	455.05 (455.05 to 455.05)	−1.59 (−1.82 to −1.36)*
85 + years	71,988	74,954	4.12	1732.97 (1732.97 to 1732.97)	1,164.76 (1,164.76 to 1,164.76)	−1.55 (−2.86 to −0.22)*

AAPCs with statistical significance are indicated by an asterisk (*). For the age-stratified (#Age) analyses, the AAMR is calculated using the crude mortality rate, and the AAPC is also derived from the crude mortality rate. AAMR, age-adjusted mortality rate; CI, confidence interval; AAPC, average annual percent change.

### 3.1 Sex stratified

**Figure 1:**
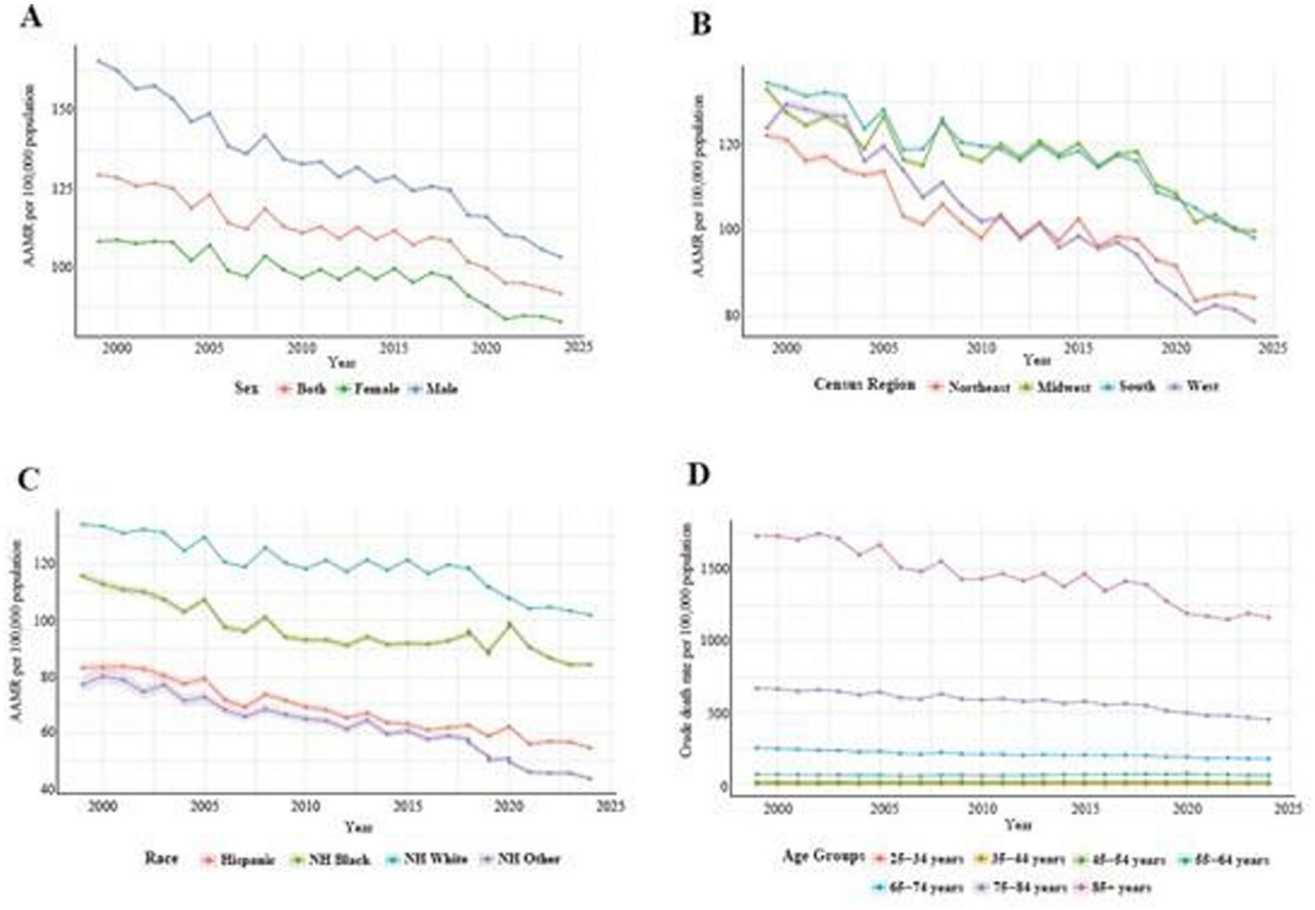
Trends in age-standardized mortality from respiratory diseases in the United States. **(A)** Age-adjusted mortality rates (AAMRs) stratified by sex. **(B)** AAMRs stratified by U.S. Census region (Northeast, Midwest, South, and West). **(C)** AAMRs stratified by race/ethnicity (Hispanic, non-Hispanic White, non-Hispanic Black, and non-Hispanic Other). **(D)** Age-specific crude mortality rates stratified by age group. Solid lines represent observed mortality rates, and joinpoint regression lines illustrate temporal trends with statistically significant changes in slope.

After stratification by sex (Figure 1A), mortality rates declined significantly in both males and females. Among males, deaths increased from 110,218 in 1999 to 129,668 in 2024 (+17.65%), while AAMR decreased from 165.44 to 103.36 per 100,000 (AAPC −1.91%). Among females, deaths rose from 117,767 to 133,541 (+13.39%), with AAMR declining from 108.25 to 82.87 per 100,000 (AAPC −1.13%). Throughout the study period, males consistently exhibited higher mortality rates than females.

Segmented analyses revealed distinct temporal patterns. In males, AAMR declined rapidly from 1999 to 2007 (APC − 2.23%), followed by a slower decrease during 2007–2018 (APC − 1.09%), and a more pronounced decline after 2018 (APC − 2.97%). In females, AAMR showed a modest reduction from 1999 to 2017 (APC − 0.69%), followed by a sharper decline from 2017 to 2024 (APC − 2.24%).

Overall, although mortality rates declined in both sexes, the reduction was more substantial among males, while absolute deaths increased in both groups, indicating that improvements in age-adjusted mortality have not translated into reduced mortality burden.

### 3.2 Census region stratified

When stratified by U.S. Census region (Figure 1B), age-adjusted mortality rates (AAMR) declined across all regions. The West showed the greatest reduction (AAPC −1.77, 95% CI − 2.65 to −0.89) and the lowest AAMR in 2024 (78.84 per 100,000), whereas the Midwest had the slowest decline (AAPC −1.04, 95% CI − 1.39 to −0.69) and the highest AAMR (99.86 per 100,000). The Northeast and South also experienced significant declines (AAPC −1.57 and −1.20%, respectively). Despite these improvements, absolute deaths increased in all regions except the Northeast, with the largest rise in the South (29.74%), indicating that declining age-adjusted risks have not translated into reduced mortality burden.

Segmented analyses revealed heterogeneous temporal patterns. In the Northeast, AAMR declined from 1999 to 2010 (APC − 1.88%), plateaued during 2010–2015, and then decreased more rapidly after 2015 (APC − 2.22%). The Midwest and South showed modest declines before 2018, followed by accelerated reductions thereafter (Midwest: −0.51 to −2.70%; South: −0.81 to −2.45%). In contrast, the West exhibited greater variability, including an early stable period, a sustained decline from 2001 to 2012, a plateau from 2012 to 2017, and a sharp decrease during 2017–2021 (APC − 4.17%), followed by a weakened trend after 2021.

Overall, regional disparities persisted, with consistently higher mortality in the Midwest and South, while the West achieved the most rapid improvements. However, increasing absolute deaths in several regions underscore the growing population burden despite declining rates.

### 3.3 Race stratified

When stratified by race/ethnicity (Figure 1C), AAMR declined across all groups, although baseline levels and rates of decline differed markedly. Non-Hispanic White (NH White) individuals consistently had the highest mortality, decreasing from 134.03 per 100,000 in 1999 to 101.92 in 2024, followed by non-Hispanic Black (NH Black) individuals (115.76 to 84.40). In contrast, Hispanic and non-Hispanic Other (NH Other) populations exhibited lower AAMR and faster declines, with NH Other showing the greatest reduction (AAPC −2.43%) and Hispanic individuals also declining steadily (AAPC −1.69%).

Temporal patterns varied substantially. Hispanic individuals experienced a continuous decline without inflection points. NH Black populations showed a decline from 1999 to 2011, a plateau during 2011–2020, and a renewed decrease after 2020. NH White populations had a gradual decline until 2018, followed by a more rapid reduction thereafter. NH Other populations exhibited the steepest late-period decline, accelerating from −1.75% (1999–2017) to −4.16% (2017–2024), the fastest among all groups.

Despite declining AAMR, absolute deaths increased across all racial/ethnic groups, particularly among Hispanic (144.74%) and NH Other populations (173.71%), indicating that reductions in age-adjusted mortality have not translated into decreased overall mortality burden.

### 3.4 Age group stratified

When stratified by age (Figure 1D), respiratory disease mortality showed a pronounced age gradient, increasing markedly with advancing age. Using age-specific crude mortality rates, the ≥85 and 75–84 age groups had the highest mortality in 1999 (1,732.97 and 671.69 per 100,000), declining to 1,164.76 and 455.05 by 2024, yet remaining substantially higher than other groups. The greatest reductions were observed among individuals aged ≥65 years, with AAPCs of −1.44% (65–74), −1.59% (75–84), and −1.55% (≥85). In contrast, younger groups showed limited improvement, and the 25–34 group exhibited a modest but persistent increase (AAPC 0.66%). Absolute deaths increased in most age groups, except for a slight decrease in those aged 35–44 (−4.39%), with the largest rise in the 55–64 group (56.89%).

Segmented analyses revealed heterogeneous temporal patterns. Mortality in the 25–34 group increased steadily throughout the study period, while rates in the 35–44 group remained stable. Among individuals aged 45–54, mortality increased until 2018 and then declined sharply thereafter. The 55–64 group showed a complex pattern of early decline, mid-period increase, and recent reduction after 2020. In contrast, older age groups exhibited consistent overall declines with varying rates, including accelerated reductions after 2017 in the 65–84 groups. Among those aged≥85 years, mortality declined steadily until 2018, then plateaued after 2021.

Overall, older adults bore the highest mortality burden and experienced the greatest improvements, whereas younger groups showed limited progress, highlighting emerging disparities in mortality trends.

### 3.5 Sex-census region cross-analysis

**Figure 2:**
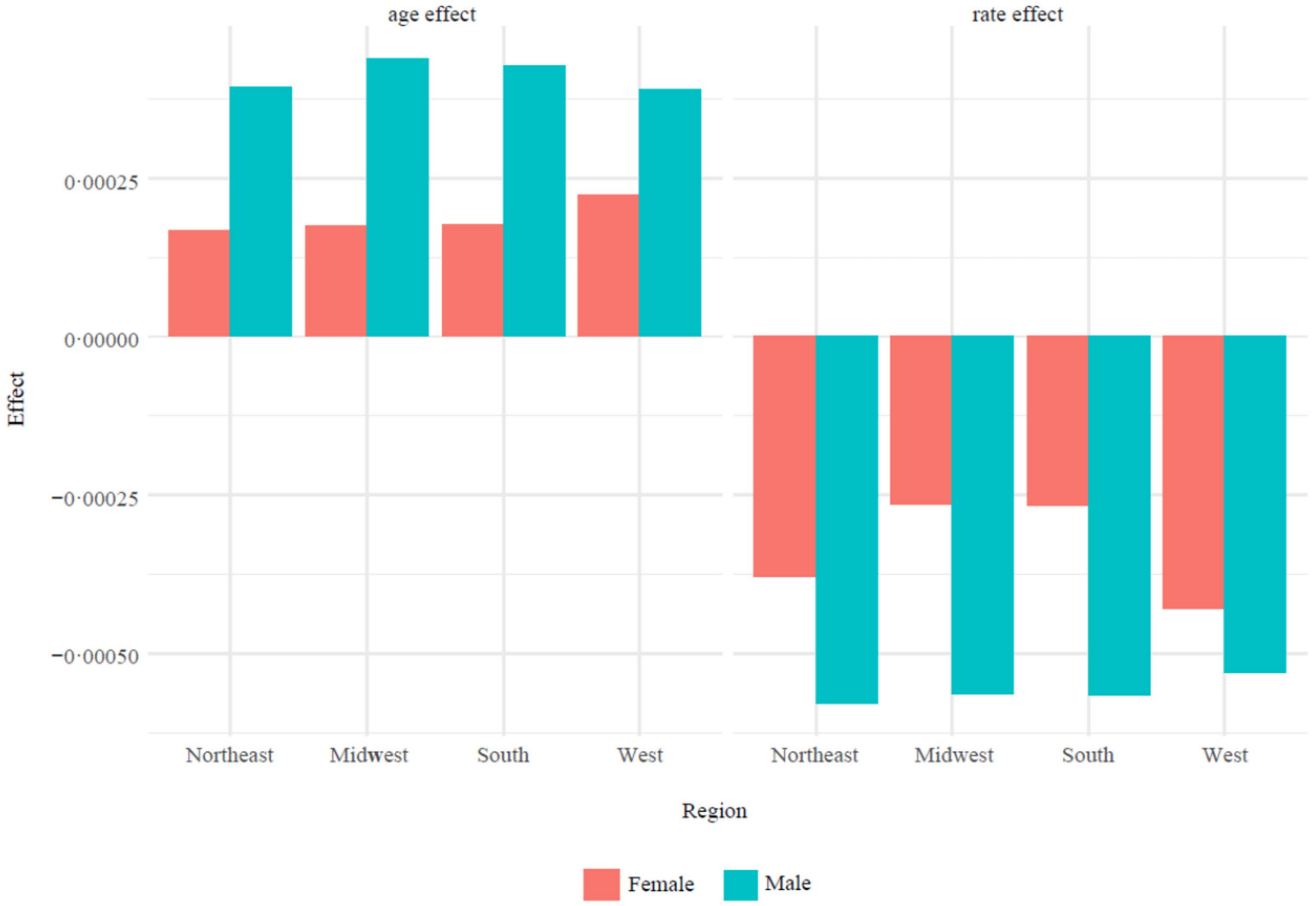
Cross-sectional analysis of respiratory system mortality by sex and region.

The age effect reflects changes in mortality attributable to population aging, examining how mortality varies under the same overall mortality level when the population structure is altered. The rate effect reflects changes in mortality resulting from variations in the underlying risk of death, such as those driven by policy interventions, advances in medical care, and other related factors. The analysis (Figure 2) showed that the age effect was positive in all regions, indicating that population aging remains one of the contributing factors to mortality from respiratory diseases. However, its overall impact on respiratory disease mortality may be relatively modest, and this finding warrants further investigation. The rate effect was negative across all age groups, indicating a declining trend in mortality from respiratory diseases and a reduction in mortality risk in all regions. However, notable regional differences in respiratory disease mortality still remain.

### 3.6 State-level distribution

**Figure 3:**
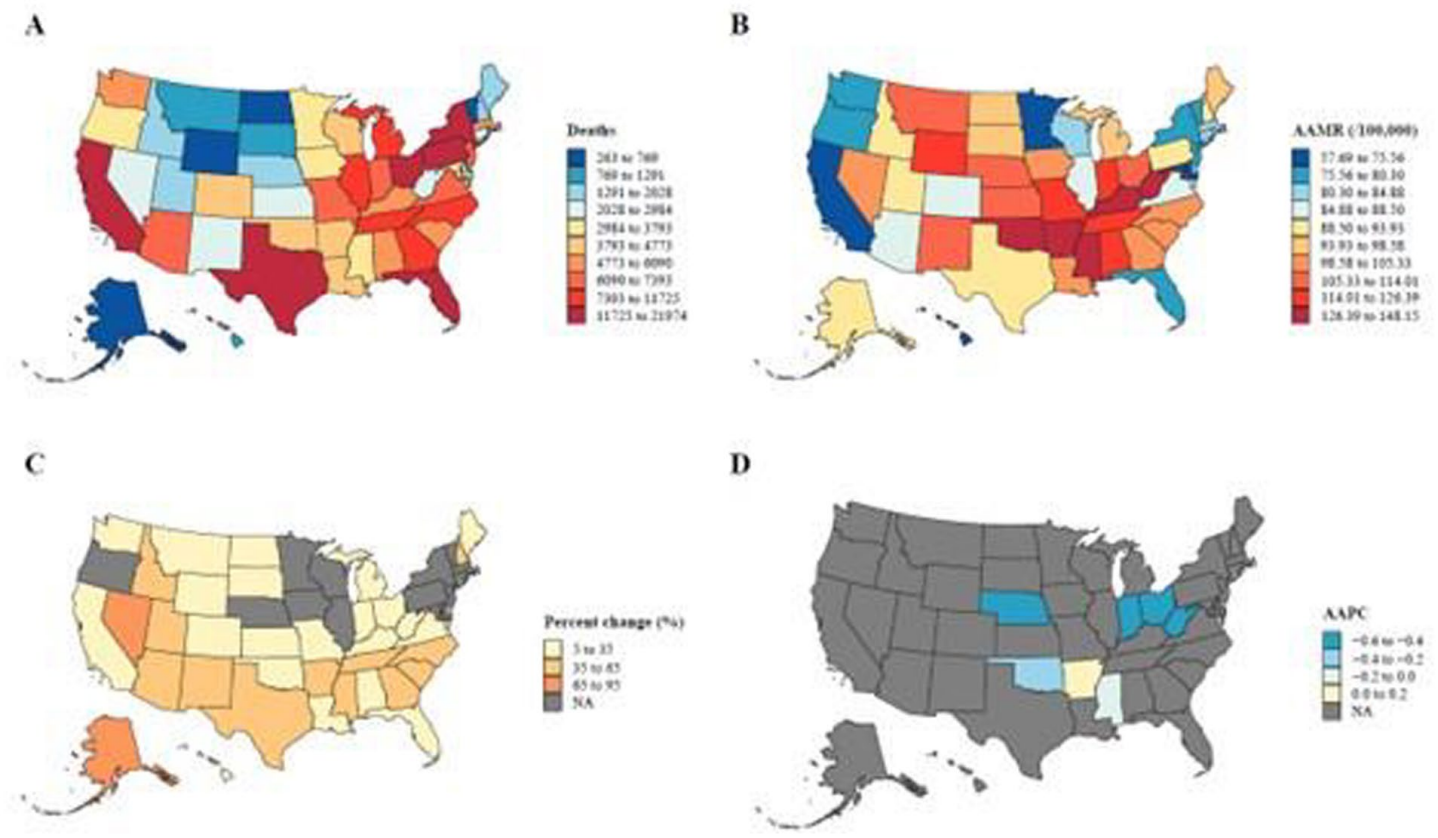
Spatial distribution at the state level. **(A)** Absolute number of respiratory disease deaths by state in 2024. **(B)** Age-adjusted mortality rates (AAMRs) per 100,000 population by state in 2024. **(C)** Percent change in the number of deaths between 1999 and 2024. **(D)** Average annual percent change (AAPC) in mortality rates estimated using Joinpoint regression.

State-level spatial analyses revealed substantial geographic heterogeneity in respiratory disease mortality across the United States. Absolute deaths were concentrated in populous states such as Texas, California, and Florida, whereas less populous regions, including parts of the Mountain West and New England, had fewer deaths (Figure 3A). In contrast, AAMR showed a distinct clustering pattern, with higher rates in the South, Appalachian region, and parts of the Midwest, while the West Coast, Northeast, and several Mountain states exhibited lower levels. This spatial distribution aligns with regional trends, where the South and Midwest consistently maintained higher mortality, and the Northeast and West showed lower levels and more pronounced declines (Figure 3B).

Despite declining AAMR nationwide, the absolute number of deaths increased in most states (Figure 3C). National deaths rose from 227,985 in 1999 to 263,209 in 2024 (+15.45%), with most states experiencing increases of 5–65% and a few reaching up to 95%. These patterns suggest that reductions in age-adjusted mortality risk have not translated into decreased mortality burden, likely reflecting population growth and aging, particularly among older adults who bear the highest mortality risk.

State-level AAPC distributions further indicated uneven progress (Figure 3D), with most states showing modest declines and a few approaching stability, highlighting spatial inequities in mortality reduction. Combined with sex- and race/ethnicity-specific patterns, these findings underscore persistent disparities across both geographic and population subgroups.

## 4 Discussion

This study conducted a comprehensive decomposition analysis of trends in respiratory disease mortality in the United States from 1999 to 2024. We found that, over the study period, the absolute number of deaths from respiratory diseases increased, whereas the mortality rate declined. This seemingly paradoxical epidemiological pattern is likely the result of the combined effects of population structural changes, advances in medical technology, environmental policy interventions, and broader socioeconomic factors. From the cross-analysis, population aging was found to exert persistent upward pressure across all regions and both sexes. Moreover, there was evident heterogeneity between sexes and across regions, indicating that the epidemiologic transition of respiratory diseases has not occurred uniformly across population subgroups.

Both the univariate and cross-analyses indicate that population aging is an important driver of the increase in the absolute number of deaths (12). Respiratory diseases, particularly COPD and pneumonia, exhibit a marked increase in incidence and mortality risk with advancing age. According to data from the United States Census Bureau, the proportion of the population aged 65 years and older increased substantially between 1999 and 2024 (13). Respiratory diseases are highly concentrated among older adults, and the large size of the elderly population substantially elevates the overall number of deaths. At the same time, the COVID-19 pandemic from 2019 to 2023 had a substantial impact on respiratory disease mortality in the United States. The marked increase in COVID-19-related deaths between 2020 and 2021 may have indirectly exacerbated the overall burden of respiratory diseases (14). This likely occurred through indirect pathways, such as disruptions to routine healthcare services, delayed diagnosis and treatment of pre-existing respiratory conditions, and potential increases in exposure to risk factors (15). The decline in mortality may be related to ongoing improvements in the healthcare system and the effective implementation of public health policies. Several public health and healthcare system changes occurred during the study period and may provide important context for the observed mortality trends. In clinical practice, evidence-based guidelines have been widely adopted, promoting standardized and individualized management of COPD and asthma. In 2009, the United States enacted *the Family Smoking Prevention and Tobacco Control Act*, which granted the Food and Drug Administration (FDA) regulatory authority over tobacco products (16). At the same time, the increase in influenza and pneumococcal vaccination coverage has effectively prevented fatal lower respiratory tract infections, which may be a factor contributing to the decline in mortality. In addition, policies such as *the Affordable Care Act* (ACA) have expanded health insurance coverage, enabling a larger proportion of low-income populations to obtain early diagnosis and continuous treatment (17). Improvements in ambient air quality constitute another external determinant, which may have jointly contributed to better respiratory health outcomes (18). During this period, the U.S. Environmental Protection Agency (EPA) repeatedly tightened *the National Ambient Air Quality Standards* (NAAQS), establishing more stringent limits for major air pollutants such as ozone (O₃) and fine particulate matter (PM₂.₅) (19). However, because our ecological trend analysis was not designed to evaluate the causal effects of specific policies, these temporal associations should be interpreted as contextual observations rather than direct evidence of policy effectiveness.

Overall, mortality rates were higher in men than in women, indicating a greater risk of death from respiratory diseases among males. This pattern may be partly attributable to historical differences in smoking behaviors between sexes. In the United States, although the gap in smoking prevalence between men and women has been narrowing, the cumulative pack-years of smoking remain substantially higher among older men (20); for example, men are more likely to develop COPD with predominant emphysema (21). In addition, occupational exposure is another key social determinant. Traditionally, men have been more likely to work in occupations such as mining, construction, and manufacturing, which are characterized by high levels of dust and chemical exposure; this substantially increases their risk of developing pneumoconiosis, occupational asthma, and COPD (22).

The cross-analysis by sex and region showed that population aging made a positive contribution in all sex–region strata, further reflecting the established trend toward population aging in the United States. Notably, in all regions, the aging effect was consistently greater in men than in women. This pattern may reflect a higher baseline mortality risk among men, which is consistent with the findings from the sex-specific analyses. Previous studies have demonstrated that mortality from respiratory diseases has consistently been higher in men than in women, which may further amplify the impact of population aging on population-level mortality (23). In contrast to the aging effect, age-specific mortality rates declined for both sexes across all regions, indicating substantial progress in disease prevention, early detection, and clinical management. Importantly, the magnitude of this decline was consistently greater in men than in women. This finding may reflect differences in the timing and intensity of exposure to key risk factors, particularly tobacco use.

The contributions of both population aging and declining mortality rates were relatively greater in the Southern and Midwestern United States, suggesting higher baseline risks in these regions. Relevant literature indicates that the chemical composition of particulate matter in the Midwestern United States is relatively distinctive, being dominated by secondary inorganic aerosols (24). These components are generated by reactions between ammonia emitted from fertilizers and livestock in agricultural activities and sulfur dioxide released from coal-fired power plants (25). Historically, these areas have been characterized by higher smoking prevalence, more severe environmental and occupational exposures, and more limited access to healthcare resources, all of which have contributed to an elevated burden of respiratory diseases (26). At the same time, the relatively larger negative rate effects observed in these regions may indicate greater potential for improvement as public health interventions and healthcare accessibility continue to expand. By contrast, both the magnitude of the age effect and the rate effect were relatively smaller in the Northeast and Western United States, which may reflect lower baseline risks and a more stable epidemiological profile.

The study found that the average annual percent change in the 25–34-year age group (+0.66%) was increasing, representing the only significantly rising age group in the entire table. This finding raises concern that mortality from respiratory diseases in the United States may be exhibiting a trend toward younger ages. From the perspective of the disease spectrum, asthma, as one of the major chronic respiratory diseases, is particularly prominent as a cause of death in younger populations. A study published in the *Annals of Allergy, Asthma & Immunology* analyzed asthma mortality data in the United States from 2000 to 2019 and found that, although asthma mortality rates declined in the overall population, the proportion of deaths occurring in the home environment did not improve (27). More importantly, the peak age of mortality from asthma is much lower than that of COPD and other diseases, with a large proportion of fatal acute exacerbations occurring in children, adolescents, and young to middle-aged adults. This pattern may render asthma a key condition driving the “younger-age” shift in respiratory disease mortality.

## 5 Conclusion

The findings of this study indicate that trends in respiratory disease mortality in the United States are shaped by the dynamic interplay between population aging and improvements in age-specific mortality, with substantial variation by sex and geographic region. Although meaningful progress has been made in reducing mortality, sustained efforts are still required to address demographic pressures and persistent disparities. Specifically, population aging will continue to exert upward pressure on respiratory disease mortality, underscoring the need for targeted strategies for older adults; the observed sex differences suggest that interventions should be tailored to the distinct risk profiles and epidemiologic trajectories of men and women; and the marked regional heterogeneity indicates that geographically targeted policies may help narrow gaps in respiratory health outcomes and further improve population health.

### 5.1 Limitations

This study has certain limitations. First, the analysis was based on aggregated data from the CDC WONDER database and did not incorporate individual-level risk factors such as smoking history, socioeconomic status, or comorbidities. Second, the decomposition approach assumes independence between population structure and mortality rates, which may not fully capture the complex interactions between demographic and epidemiologic factors.

All authors are from a School of Humanities and Social Sciences, not a clinical pulmonary medicine department. While this background offers valuable social and behavioral insights into respiratory diseases, it may introduce bias or incomplete understanding of clinical pathophysiology and practice.

## Data Availability

The original contributions presented in the study are included in the article/Supplementary material, further inquiries can be directed to the corresponding author.
