# Supplementary material for: Trends in respiratory disease mortality in the United States, 1999–2024
Source: Front Med (Lausanne). 2026 Jul 23;13:1879935. doi: 10.3389/fmed.2026.1879935 (PMC13442642; doi:10.3389/fmed.2026.1879935)
Supplement: Supplementary file 1 [file Data_Sheet_2.PDF]

The APCs and 95% CIs by Joinpoint segments for each group

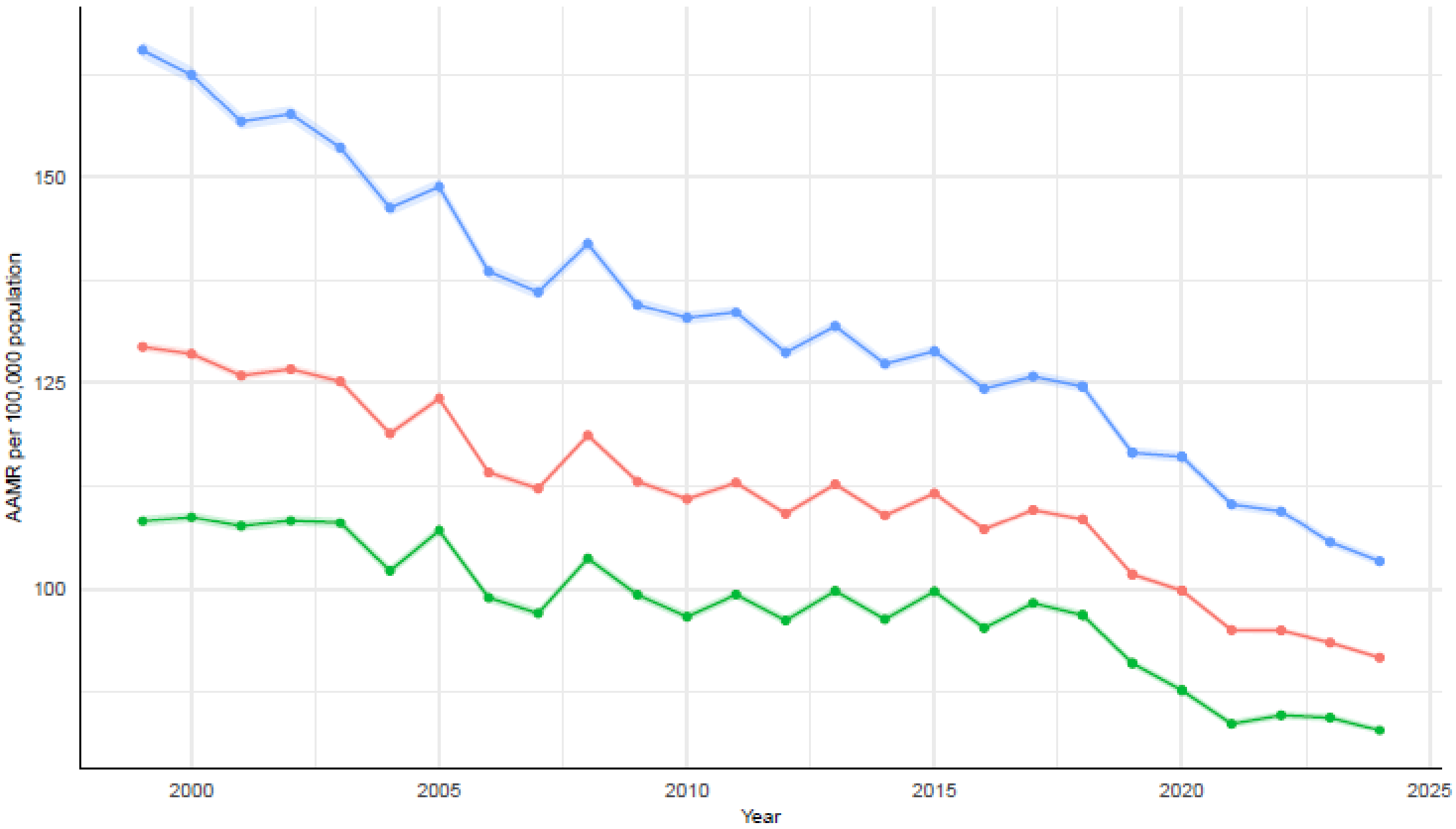

Sex

Both: 1999 to 2018 APC: −1.00 (−1.20 to −0.81); *2018 to 2024 APC: −2.52 (−3.58 to −1.44)*

Female: 1999 to 2017 APC: −0.69 (−0.94 to −0.44); *2017 to 2024 APC: −2.24 (−3.22 to −1.26)*

Male: 1999 to 2007 APC: −2.23 (−2.77 to −1.69); *2007 to 2018 APC: −1.09 (−1.46 to −0.73)*; 2018 to 2024 APC: −2.97 (−3.73 to −2.21)*

A Sex stratified

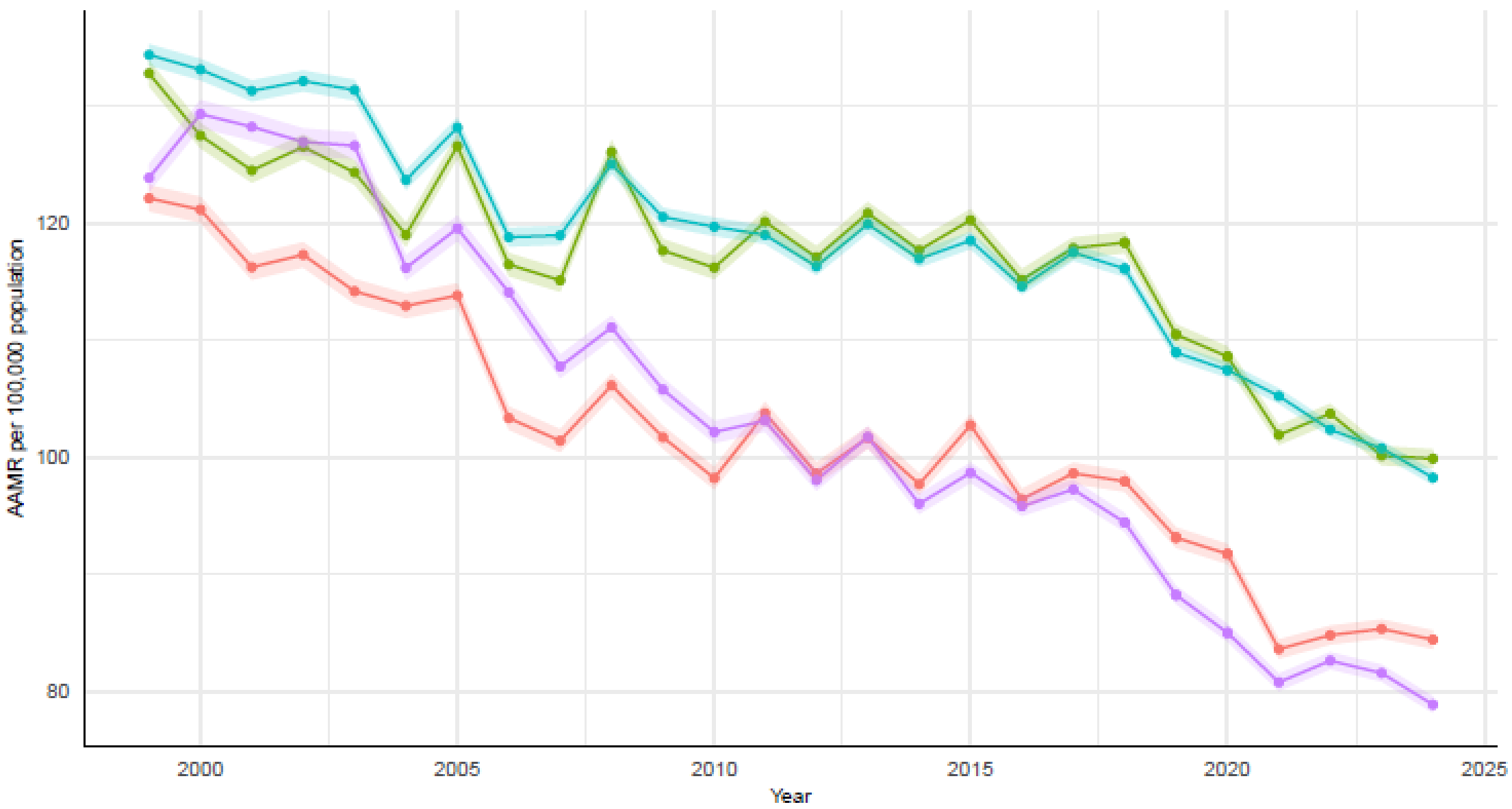

Census Region

Northeast: 1999 to 2010 APC: −1.88 (−2.41 to −1.34); *2010 to 2015 APC: 0.31 (−2.22 to 2.90); 2015 to 2024 APC: −2.22 (−2.93 to −1.50)*

Midwest: 1999 to 2018 APC: −0.51 (−0.75 to −0.27); *2018 to 2024 APC: −2.70 (−4.01 to −1.36)*

South: 1999 to 2018 APC: −0.81 (−0.99 to −0.63); *2018 to 2024 APC: −2.45 (−3.39 to −1.50)*

West: 1999 to 2001 APC: 1.77 (−5.19 to 9.25); 2001 to 2012 APC: −2.42 (−2.94 to −1.90); *2012 to 2017 APC: −0.38 (−2.45 to 1.73); 2017 to 2021 APC: −4.17 (−7.26 to −0.99)*; 2021 to 2024 APC: −0.76 (−4.00 to 2.60)

B Census region stratified

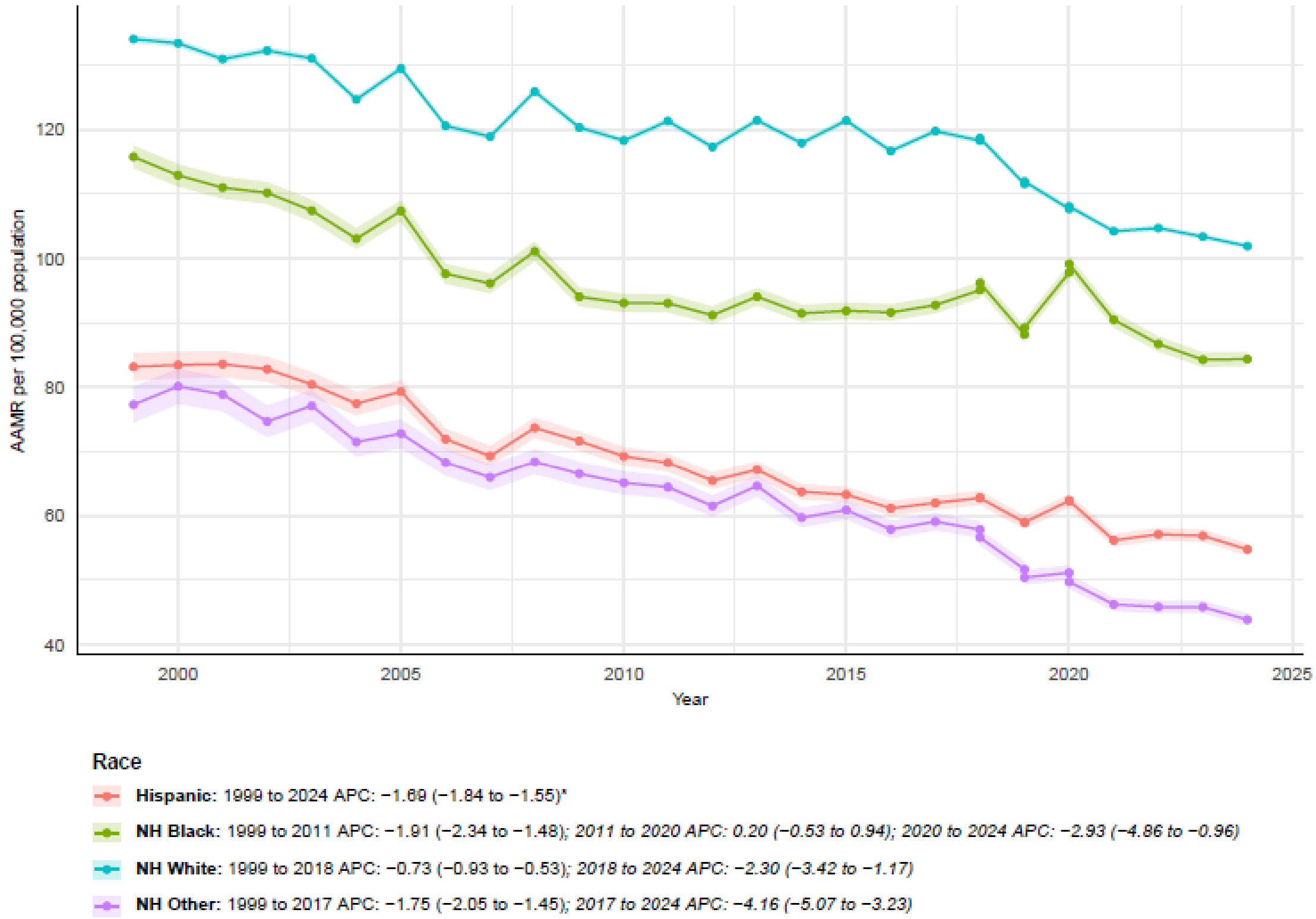

C Race stratified

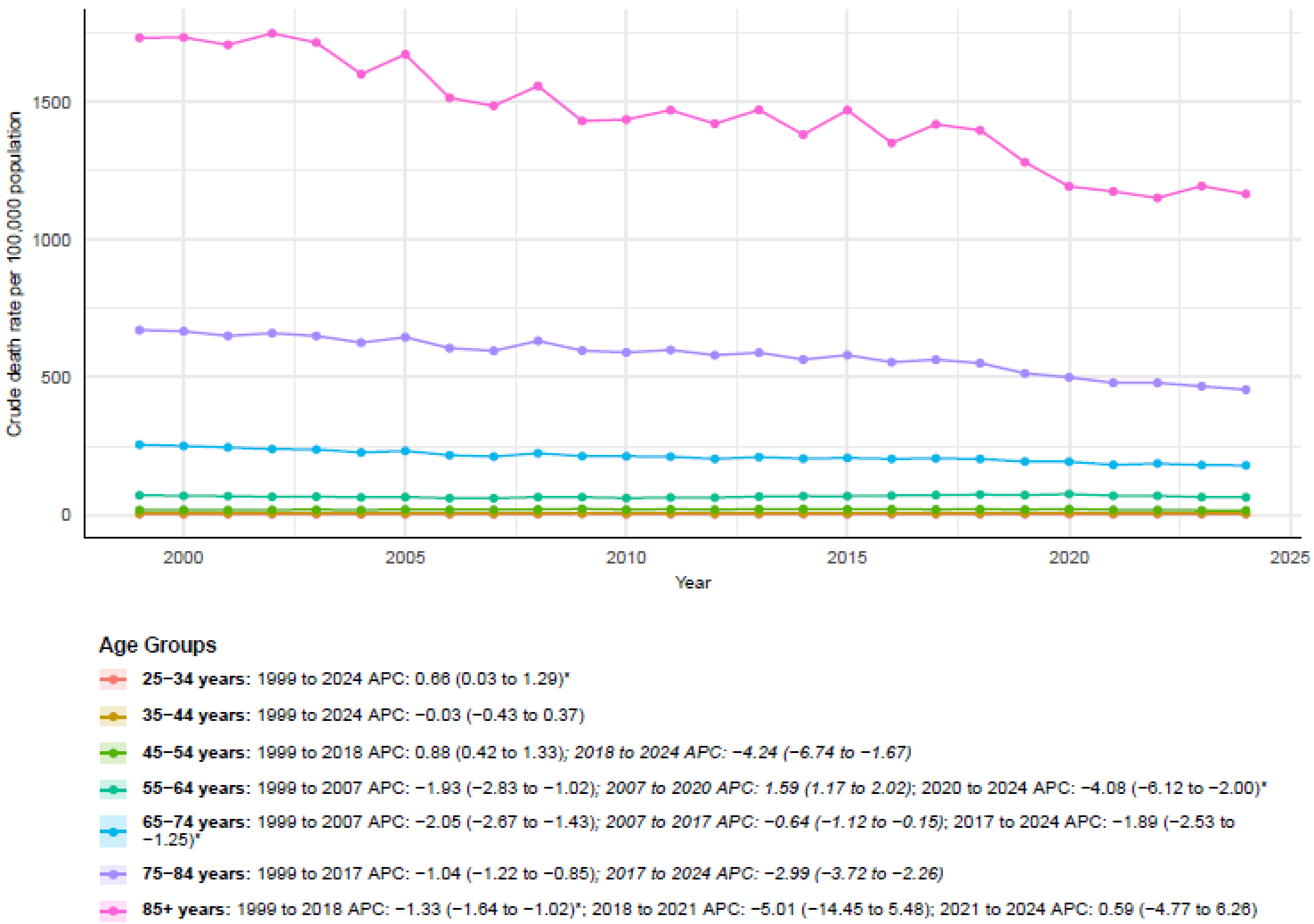

D Age group stratified
